# Prevalence of Dental Caries and Associated Factors in East Africa, 2000–2020: Systematic Review and Meta-Analysis

**DOI:** 10.3389/fpubh.2021.645091

**Published:** 2021-04-29

**Authors:** Amare Teshome, Abebe Muche, Biruk Girma

**Affiliations:** ^1^Department of Dentistry, School of Medicine, College of Medicine and Health Science, University of Gondar, Gondar, Ethiopia; ^2^Department of Human Anatomy, School of Medicine, College of Medicine and Health Science, University of Gondar, Gondar, Ethiopia

**Keywords:** dental caries, DMFT, prevalence, oral health, tooth decay

## Abstract

**Background:** Dental caries affects mastication, growth and development, and school attendance and has a long-term psychological effect on affected individuals. In developing countries, the prevalence of dental caries is increasing due to the growing consumption of sugary foods, poor tooth brushing habits, and a low level of awareness about dental caries. Even if there was a high prevalence of dental caries in sub-Saharan Africa, there is a paucity of data on the prevalence of dental caries in East Africa. Hence, this study aimed to determine the prevalence of dental caries and associated factors in East Africa.

**Methods:** A systematic search of articles was conducted in MEDLINE, Scopus, and Google Scholar using all the synonyms of dental caries in published literature (until December 2020) in East Africa. Important data were extracted using a standardized data extraction form prepared in Excel. Stata software (version 14.0) was used to calculate the pooled prevalence of dental caries. Besides, subgroup analysis was done based on country and dentition type. Moreover, associated factors of dental caries were assessed and the overall effect was presented in the form of odds ratios. The quality of the included studies was evaluated using the Joanna Briggs Institute reviewers' manual.

**Results:** The overall pooled prevalence of dental caries was found to be 45.7% (95% CI = 38.0–53.4). The pooled prevalence was high in Eritrea (65.2%, 95% CI = 49.2–81.1), followed by Sudan (57.8%, 95% CI = 36.0–79.7), and a low prevalence was found in Tanzania (30.7%, 95% CI = 21.5–39.9). Moreover, the subgroup analysis revealed a prevalence of 50% (95% CI = 38.4–62.1) in permanent dentition and 41.3% (95% CI = 33.5–49.2) in mixed dentition. The overall mean decayed, missed, and filled permanent (DMFT) and primary (dmft) teeth were 1.941 (95% CI = 1.561–2.322) and 2.237 (95% CI = 1.293–3.181), respectively. High DMFT scores were reported in Sudan (3.146, 95% CI = 1.050–5.242) and Uganda (2.876, 95% CI = 2.186–3.565). Being female (OR = 1.34, 95% CI = 1.24–1.46) and having poor tooth brushing habit (OR = 1.967, 95% CI = 1.67–2.33) were independent risk factors of dental caries.

**Conclusion:** The overall prevalence of dental caries was comparatively high. Being female and poor oral health practice were independent risk factors of dental caries. The Ministry of Health of the member countries, along with dental associations of each country, ought to offer due attention to strengthen the oral health program in schools and primary health care centers and the implementation of school water fluoridation.

## Background

Dental caries is a bacterial infectious disease that affects the calcified tissue of the tooth and causes dissolution of the organic component and demineralization of the inorganic portion (1). It is caused by bacterial plaque deposition on the surface of the tooth (2, 3), and frequent consumption of fermentable carbohydrates facilitates the progression of cavitation. Oral microbes such as *Streptococcus mutans* metabolize fermentable carbohydrates and produce lactic acid, which lowers the oral pH to a level where the minerals of dentin and enamel dissolve easily (4–7).

Dental caries is a global public health problem and affects all human race (8). The treatment cost in low-income countries alone exceeds the total child health care cost (9). This disease is found in all socioeconomic strata and affects the quality of life, school attendance, eating practices, growth, and the development of children and has psychological impacts on the performance of patients (3–5). In developing countries, dental caries remains untreated due to inappropriate, unaffordable, and unavailable dental services and to the scarcity of professionals (10). Moreover, dental caries costs US $298 billion in direct treatment costs to the global economy, 4.6% of the global health budget, and 144 billion losses due to loss of productivity (11).

In developed countries, the prevalence of dental caries is declining due to advanced dental facilities and the increased awareness of oral hygiene (10, 12–14). However, an unprecedented increase in prevalence is reported in developing countries due to the growing consumption of sugary foods, poor tooth brushing habits, and the absence of adequate dental services (10, 14–16). In developing countries, especially sub-Saharan Africa, the prevalence of dental caries varies according to the population group and socioeconomic status (12). The prevalence rates were 40.98% in Ethiopia (17), 52.4% in Sudan (18), 50.3% in Kenya (19), and 40.2% in Tanzania (20).

In East African countries, there is scarcity of data on the prevalence of dental caries. Hence, this study aimed to determine the pooled prevalence of dental caries and associated factors in East Africa.

## Methods and Materials

The results of the present review are reported according to the Meta-analysis of Observational Studies in Epidemiology (MOOSE) guideline (21).

### Inclusion and Exclusion Criteria

Studies that meet the following inclusion criteria were included in the systematic review and meta-analysis.

- Observational studies were done on the prevalence of dental caries and associated factors in East African countries.
- Data regarding dental caries in terms of proportion or DMFT/dmft
- Full-text published articles
- Studies that did not report specific outcomes quantitatively
- Abstracts, case reports, review articles, comments, posters, editorial reviews, and letters to the editor were excluded.

### Search Strategy

A systematic search of studies was carried out in MEDLINE, Scopus, and Google Scholar databases without language restriction and included studies published up to December 2020. Article search was done using key terms. Besides, the reference lists of all relevant studies were screened. The search strategy was built using a combination of keywords for the main axes of the research questions. The search strategy used key terms related to (a) dental caries, tooth decay, or DMFT and (b) East Africa countries (Ethiopia, Djibouti, Somalia, Eritrea, Kenya, Burundi, Tanzania, Sudan, South Sudan, Rwanda, and Uganda). The search terms were predefined to allow a comprehensive search that includes text fields within records and Medical Subject Headings (MeSH terms) were used to help expand the search. The MeSH terms used for Scopus were: {(Dental Caries OR caries [Mesh] OR Tooth decay OR DMFT index [Mesh] OR decayed teeth) AND (risk factors [Mesh])}.

### Study Selection

Database search results were pooled and duplicate studies were removed using Endnote and manually. After the duplicates were removed, the titles and abstracts were screened and studies that were irrelevant to the research question and outcome of the study were excluded. Full-text studies that have outcomes of interest were evaluated further using the inclusion criteria. Two dental surgeons (AT and BG) independently screened information at each stage. Disagreement was resolved by the involvement of a third independent reviewer (AM).

### Data Extraction and Data Items

Data such as the first author's name, country, age, study design, sample size, and the prevalence of dental caries or decayed, missed, and filled permanent (DMFT)/primary (dmft) teeth were extracted from the selected studies by two investigators (AT and BG) independently. Important data were extracted using a standardized data extraction form prepared in Excel. The pooled prevalence or DMFT/dmft was extracted. Any disagreement between the two reviewers was resolved by discussion and consensus.

Language translator was used to translate articles published in other languages, which were translated into English, and to afford conditions for data extraction. In the case of missing data, a one-time contact was attempted to obtain the missing information from the corresponding author *via* e-mail.

### Quality Assessment

The quality of evidence was assessed using the Joanna Briggs Institute (JBI) reviewers' manual for a systematic review of prevalence and incidence studies. The reviewers critically appraised the quality of the studies based on sample representativeness, participant recruitment, sample size estimation, reliability of the measurement, and the analysis of the outcomes. Studies with a quality assessment score of 50% and above were included in the review.

### Data Analysis

Stata software version 14.0 (22) was used to determine the pooled estimates. The prevalence of dental caries was determined for individual studies and then the prevalence ratio (PR) was calculated considering 95% confidence interval (CI). The analysis was performed using the random effect model (Mantel–Haenszel model) (23). The extent and the significance of variations between the selected studies were determined by calculating the heterogeneity using Higgins' *I*^2^ statistics (24). Substantial heterogeneity was considered when *P* < 0.10 for the *Q* test and *I*^2^ ≥ 50%. Publication bias was assessed using the funnel plot and Egger's test (25). Subgroup analyses were performed based on country and dentition status. Besides, a sensitivity analysis (26) was done to determine the influential studies on the overall prevalence ratio. Moreover, associated factors of dental caries were assessed and the overall effect was determined in the forms of the odds ratios.

## Results

### Study Selection

**Figure 1:**
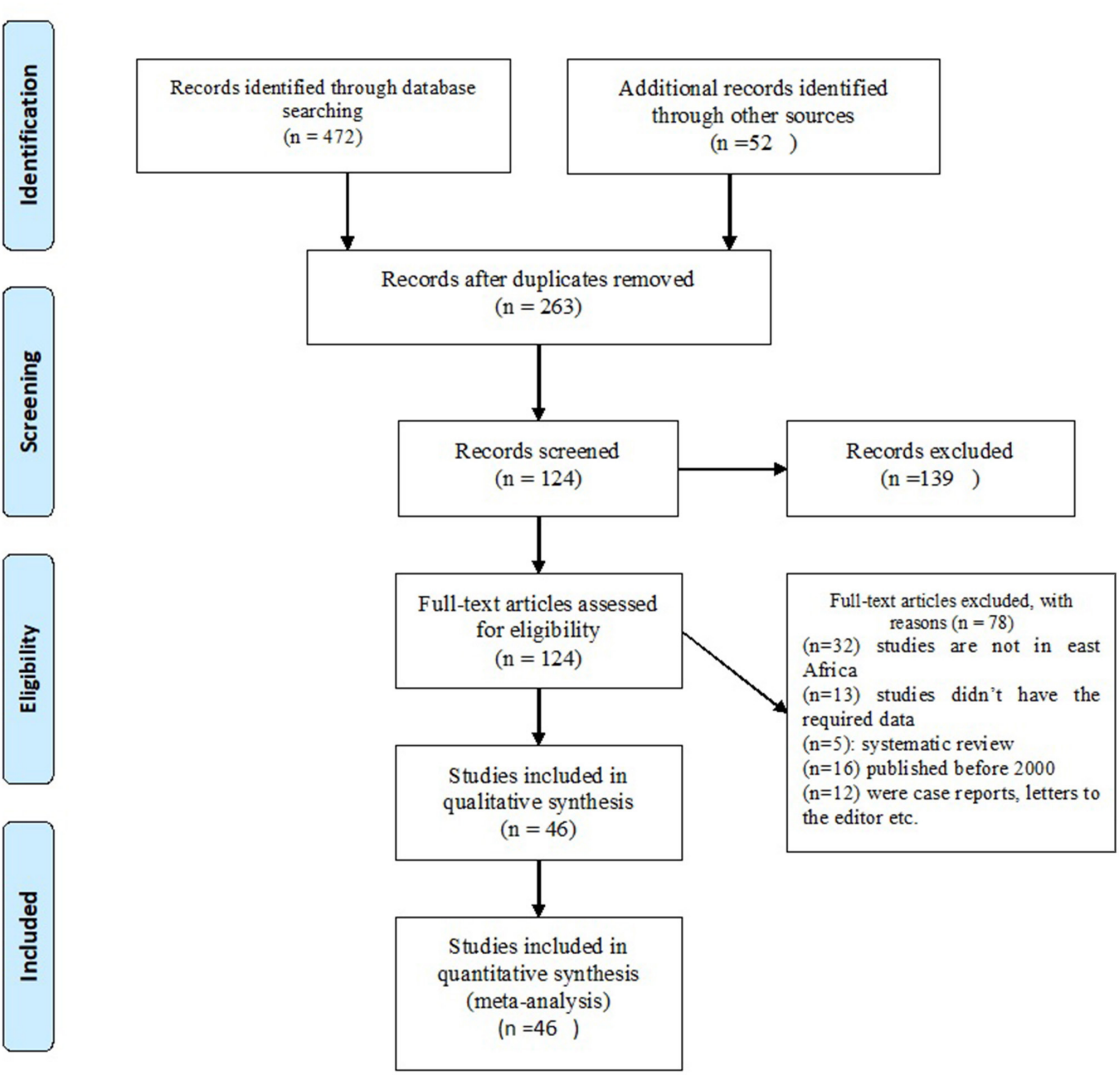
PRISMA flow diagram showing the article selection process.

As shown in the flow diagram (Figure 1), 524 studies were searched from all databases. Of which, 261 were excluded as duplicates using Endnote 7 software and manually.

The remaining 263 studies were filtered according to the titles and abstracts; 139 studies were excluded due to unrelated themes. A full-text review was done for the remaining 124 studies and identified 44 studies that meet the inclusion criteria for this review.

### Study Characteristics

Forty-six studies meet the inclusion criteria, with 27,206 population. The sample sizes of the studies ranged from 79 (27) to 1,888 (28) study participants. The studies were conducted in Ethiopia (3, 29–40), Eritrea (41–43), Tanzania (26–33), Uganda (44–50), Sudan (18, 27, 28, 51, 52), Kenya (53–58), Rwanda (59), and Somalia (60, 61). All of the included studies were conducted between 2000 and 2020 (Table 1).

**Table 1:** Study characteristics of the selected studies.

**S/No**	**References**	**Country**	**Year**	**Sample size**	**Age**	**Prevalence**	**Mean DMFT**	**Mean dmft**
1.	Walle (30)	Ethiopia	2014	422	30.31 ± 1.39	78.7%		
2.	Ayele et al. (3)	Ethiopia	2013	842	7–14 years	36.3%		
3.	Mulu et al. (31)	Ethiopia	2014	147	6–15 years	21.8%		
4.	Simon et al. (32)	Ethiopia	2003	1,736	≥12 years	21.1%		
5.	Teshome et al. (29)	Ethiopia	2016	291	12–20 years	48.5%	1.23	
6.	Berhane and Worku (33)	Ethiopia	2014	658	10–14	47.4%		
7.	Burnett et al. (2015)	Ethiopia	2015	132	6–15	74%		
8.	Rwenyonyi et al. (44)	Uganda	2011	321	18–62	57.3%	2.3	
9.	Kiwanuka et al. (45)	Uganda	2004	589	3–5	28.7%		2.4 ± 3.2
10.	Mwakatobe et al. (62)	Tanzania	2007	310	12 years	41.6%	0.76 ± 1.17	
11.	Fukuda et al. (53)	Kenya	2014	150	12 years	10%	0.24	
12.	Mafuvadze et al. (63)	Tanzania	2013	172	12 years	49.4%	0.98	
13.	Owino et al. (19)	Kenya	2010	292	12 years	50.3%	0.92 ± 50	
14.	Carneiro and Kabulwa (64)	Tanzania	2012	785	≥14 years	46.4%	1.26	
15.	Svensson et al. (60)	Somalia	2016	310	6–17	57%	2.2	2.3
16.	Wondwossen et al. (40)	Ethiopia	2004	306	2–15 years	45.3%	1.2	
17.	Kikwilu and Mandari (65)	Tanzania	2001	1,297	8–15	24%	0.41	
18.	Ahmed and Abuaffan (51)	Sudan	2015	360	6–11	47.2%	2.2	4.68
19.	Mwakayoka et al. (66)	Tanzania	2017	525	2–4 years	20.2%		
20.	Khalifa et al. (28)	Sudan	2012	1,888	≥16 years	87.7%	8.7 ± 5.9	
21.	Muwazi et al. (46)	Uganda	2005	1,092	12 years	62.5%	3.4	0.9
22.	Kutesa et al. (47)	Uganda	2015	1,978	11–13 years	42.1%	0.73,4.71	
23.	Elidrissi and Naidoo (18)	Sudan	2016	553	3–5 years	52.4%		2.3
24.	Kebede et al. (35)	Ethiopia	2012	240	29.9 ± 9.79		1.94 ± 2.12	
25.	Birungi et al. (48)	Uganda	2020	345	5–7 years	29.7%		2.1 (2.7)
26.	Mohamed Ali et al. (52)	Sudan	2017	293	7.2 ± 3.0 years	159 (54.3%)	1.3 (1.7)	3.7 (3.8)
27.	Mashoto et al. (67)	Tanzania	2009	1,745	13.8 ± 1.67 years	17.4%		
28.	Ndagire et al. (49)	Uganda	2020	406	11–19 years	66.0%	2.18 ± 2.67	
29.	Simangwa et al. (68)	Tanzania	2018	906	13.4 (±1.2)	8.8% (80)		
30.	Andegiorgish et al. (43)	Eritrea	2017	225	12 years	78%	2.5 ±2.21	
31.	Kalanzi et al. (50)	Uganda	2019	748	39 ±9.4years	83.7%	5.9 ± 5.5	
32.	Kassim and Noor (55)	Kenya	2006	141	≥18 years	43.3%	3.4	
33.	Kiwanuka et al. (45)	Uganda	2004	589	3–5 years	169 (28.7%)		2·4 ± 3·2
34.	Makhanu et al. (56)	Kenya	2009	275	13–15 years		1.54± 1.071	
35.	Masiga and M'Imunya (57)	Kenya	2013	220	*3–15 years*	65.0 %	*1.08*	*1.75*
36.	Uwayezu et al. (59)	Rwanda	2020	*226*	*7–20 years*	42.4%		
37.	Nordstrand et al. (61)	Somalia	2019	*2,093*	*4–19 years*	26%		
38.	Tagelsir et al. (27)	Sudan	2013	79	11–13-year	46.8%.	0.4 ± 0.7	1.9 ±2.8
39.	Ademe et al. (36)	Ethiopia	2020	*407*	*6–15 years*	36.9%	0.95 ± 1.57	
40.	Bogale et al. (38)	Ethiopia	2021	*1,047*	*≥18 years*	60%		
41.	Rwakatema et al. (20)	Tanzania	2015	214	27.2 ± 7.35 ddharanii 18–53 years	40.2%.	1.34 ± 2.44	
42.	Equbamichael et al. (41)	Eritrea	2006	400	*>12 years*	50%		
43.	Abdelhamid et al. (42)	Eritrea	2019	*330*	14–17 years	67.9%	2.1	
44.	Teshome et al. (37)	Ethiopia	2020	*368*	30 ± 14.766	23.64%	1.095 ± 0.24	0.13
45.	Njoroge et al. (58)	Kenya	2010	**336**	*3–5 years*	**59.5%**		2.46 ± 2.32
46.	Aynalem et al. (39)	Ethiopia	2020	*417*	12.74 (±2.556)	34.1%		

### Prevalence of Dental Caries

**Figure 2:**
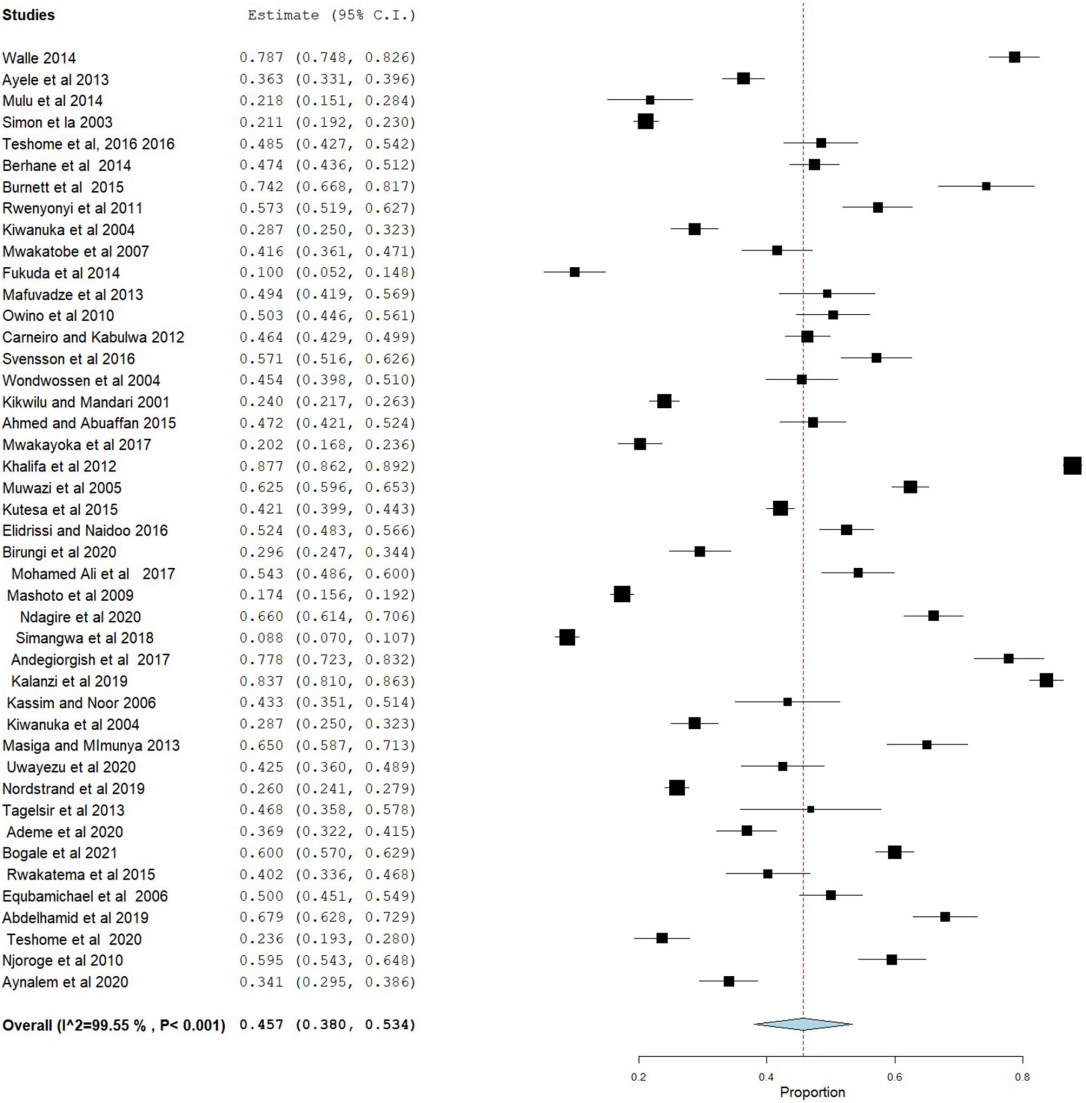
Forest plot showing the pooled prevalence of dental caries in East Africa.

**Figure 3:**
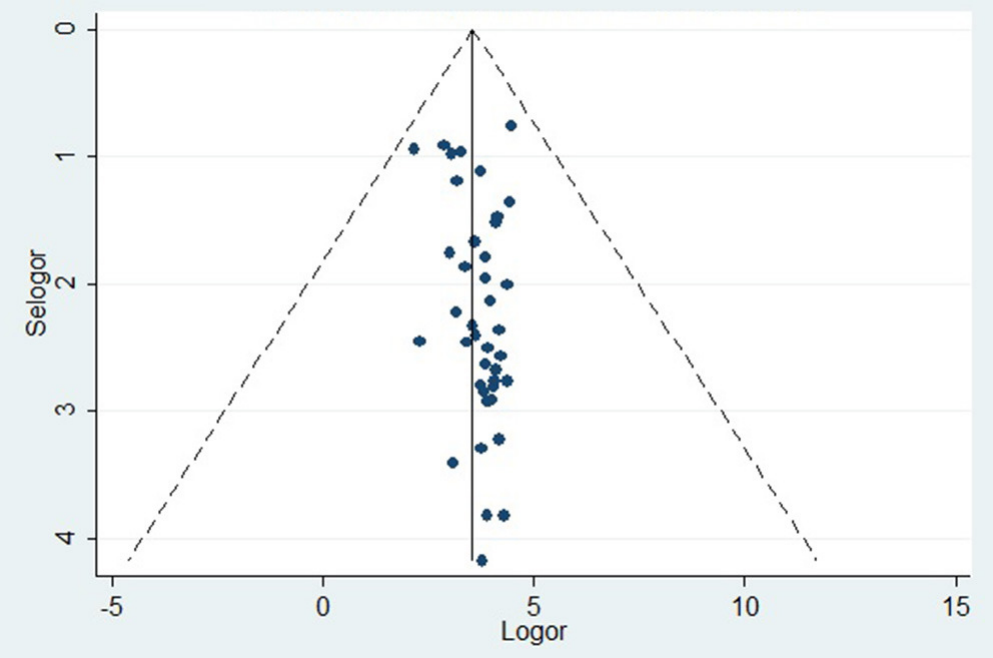
Funnel plot.

The prevalence of dental caries in this review ranged from 8.8% (68) to 87.7% (28). The DMFT of the region ranged from 0.24 (53) to 8.70 (28). Forty-four studies were included in the analysis of the pooled prevalence of dental caries. High heterogeneity was observed between the included studies, with an *I*^2^ value of 99.55%, and a random effect model was used. The overall pooled prevalence of dental caries in East Africa was found to be 45.7% (95% CI = 38.0–53.4), with heterogeneity (*I*^2^) of 99.55% (Figure 2). The funnel plot demonstrated a symmetrical distribution of the included studies (Figure 3). Moreover, the Begg's test and Egger's test showed the absence of publication bias (Pr > |*z*| = 0.398) and *P* = 0.155, respectively.

### Subgroup Analysis

**Figure 4:**
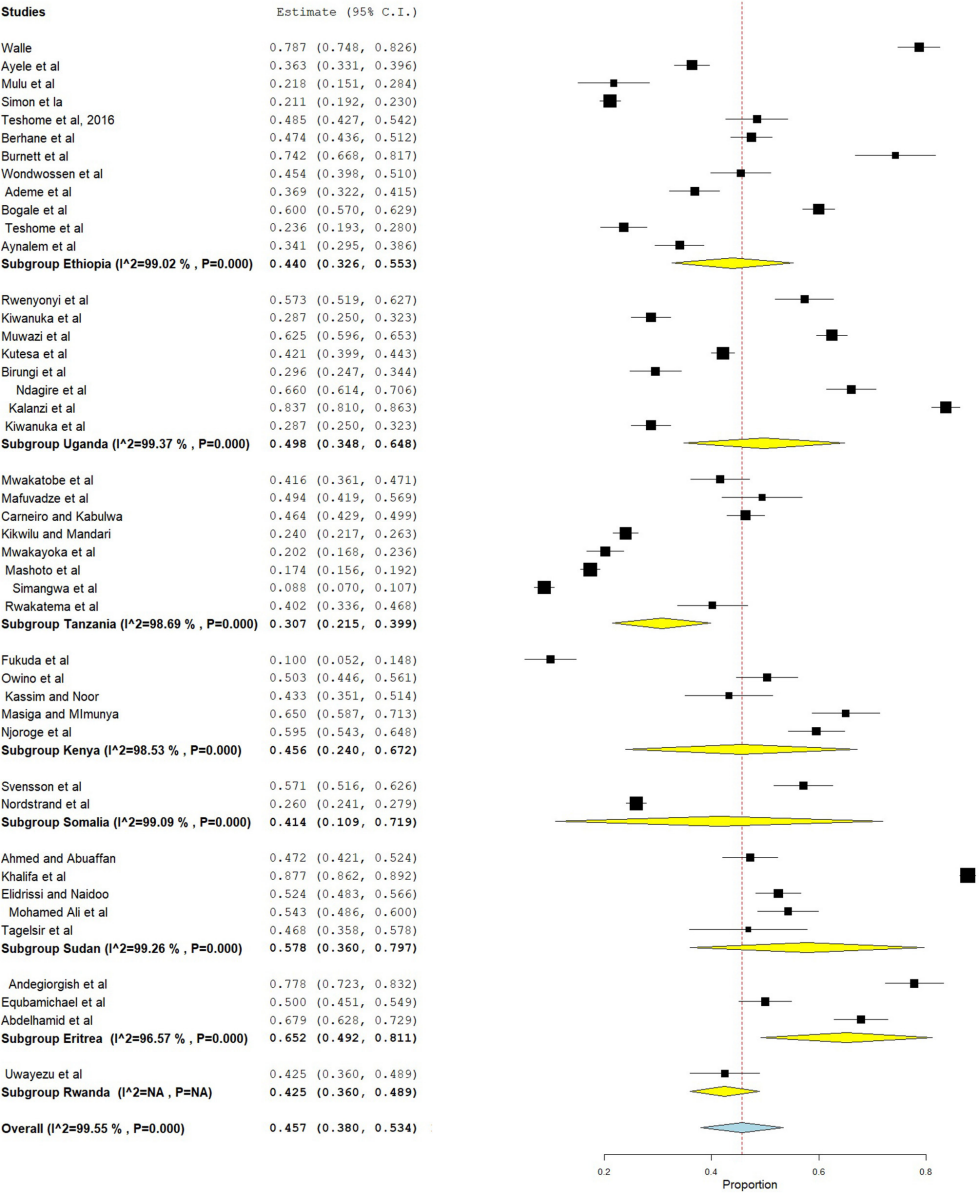
Subgroup analysis on prevalence of dental caries based on country.

**Figure 5:**
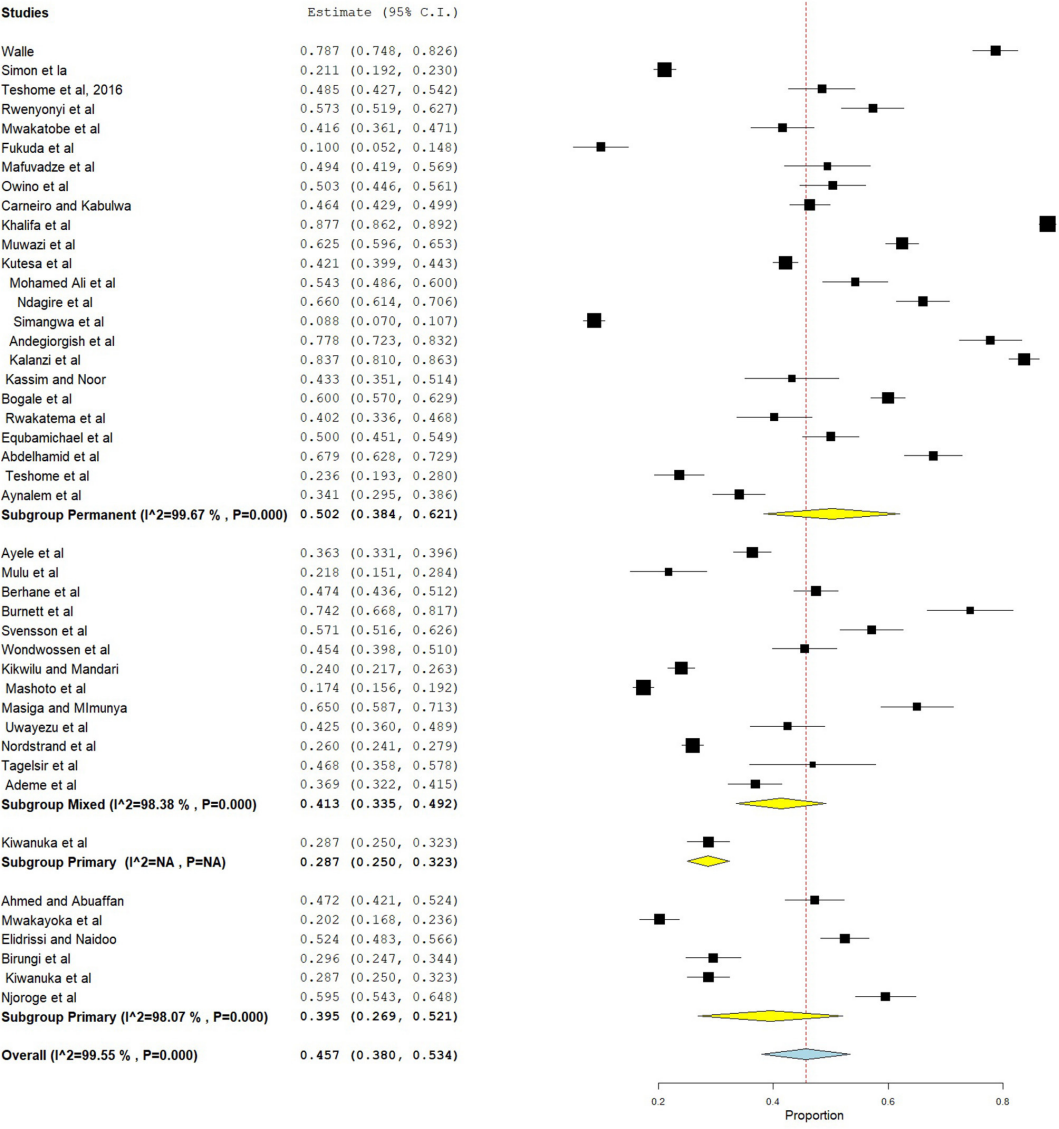
Subgroup analysis on prevalence of dental caries based on dentition status.

In this systematic review and meta-analysis, subgroup analysis was done based on country and dentition status (primary, mixed, or permanent dentition). Hence, the highest prevalence of dental caries was found in Eritrea, which was 65.2% (95% CI = 49.2–81.1), followed by Sudan (57.8%, 95% CI = 36.0–79.7) (Figure 4). Moreover, subgroup analysis revealed that the pooled prevalence of dental caries was high in those ≥12 years (50.2%, 95% CI = 38.4–62.1), followed by those 6–12 years (41.3%, 95% CI = 33.5–49.2) (Figure 5).

### Sensitivity Analysis

**Figure 6:**
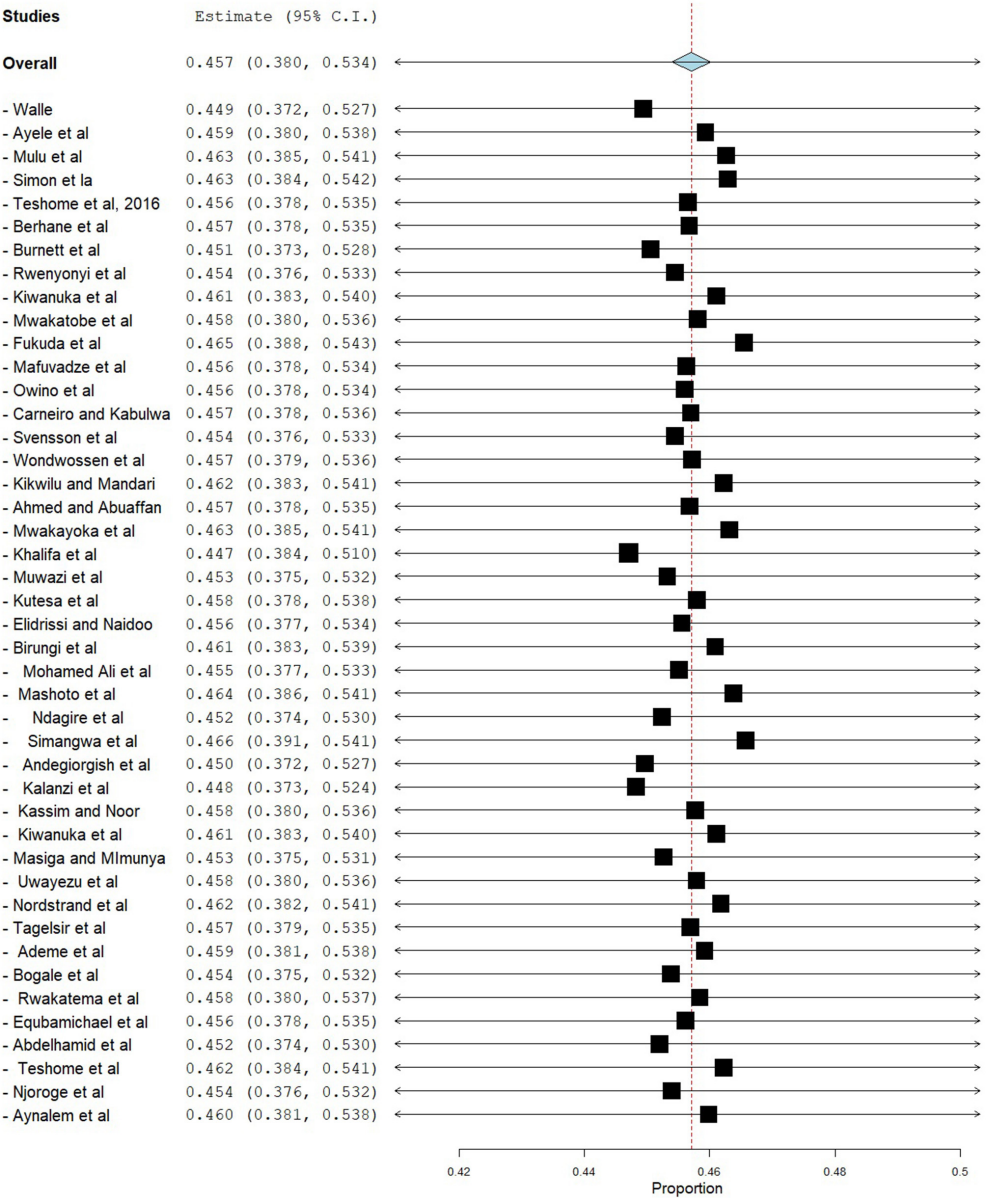
Sensitivity analysis.

Sensitivity analysis was done using the leave-one-out method to identify the source of heterogeneity and found that the pooled prevalence did not depend on the outcome of a single study. After the removal of one study stepwise, the pooled prevalence ranged from 44.7% (95% CI = 38.4–51.0) to 46.6% (95% CI = 39.1–54.1) (Figure 6).

### Decayed, Missed, and Filled Tooth and dmft

**Figure 7:**
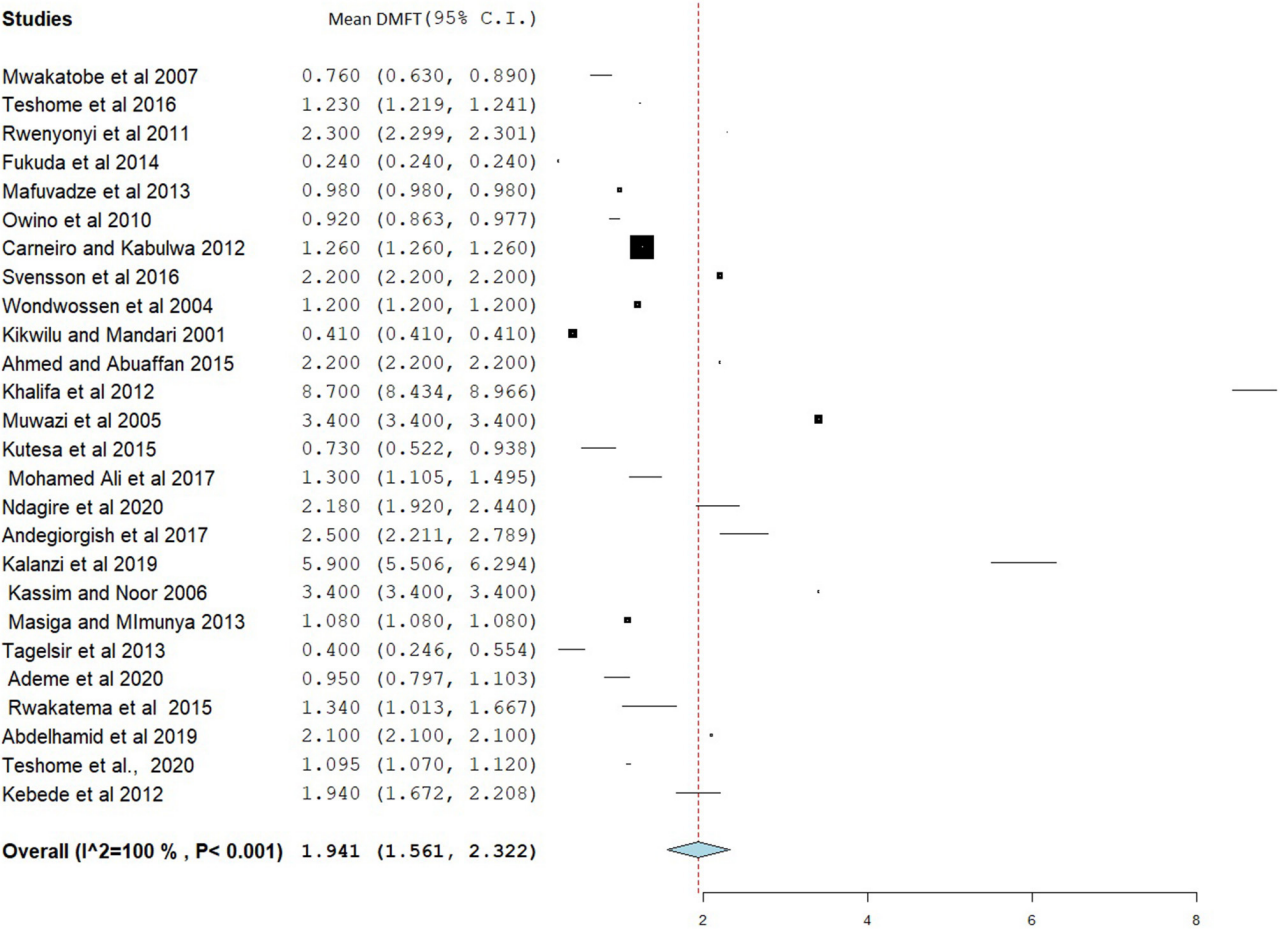
Forest plot showing the mean DMFT in East Africa.

**Figure 8:**
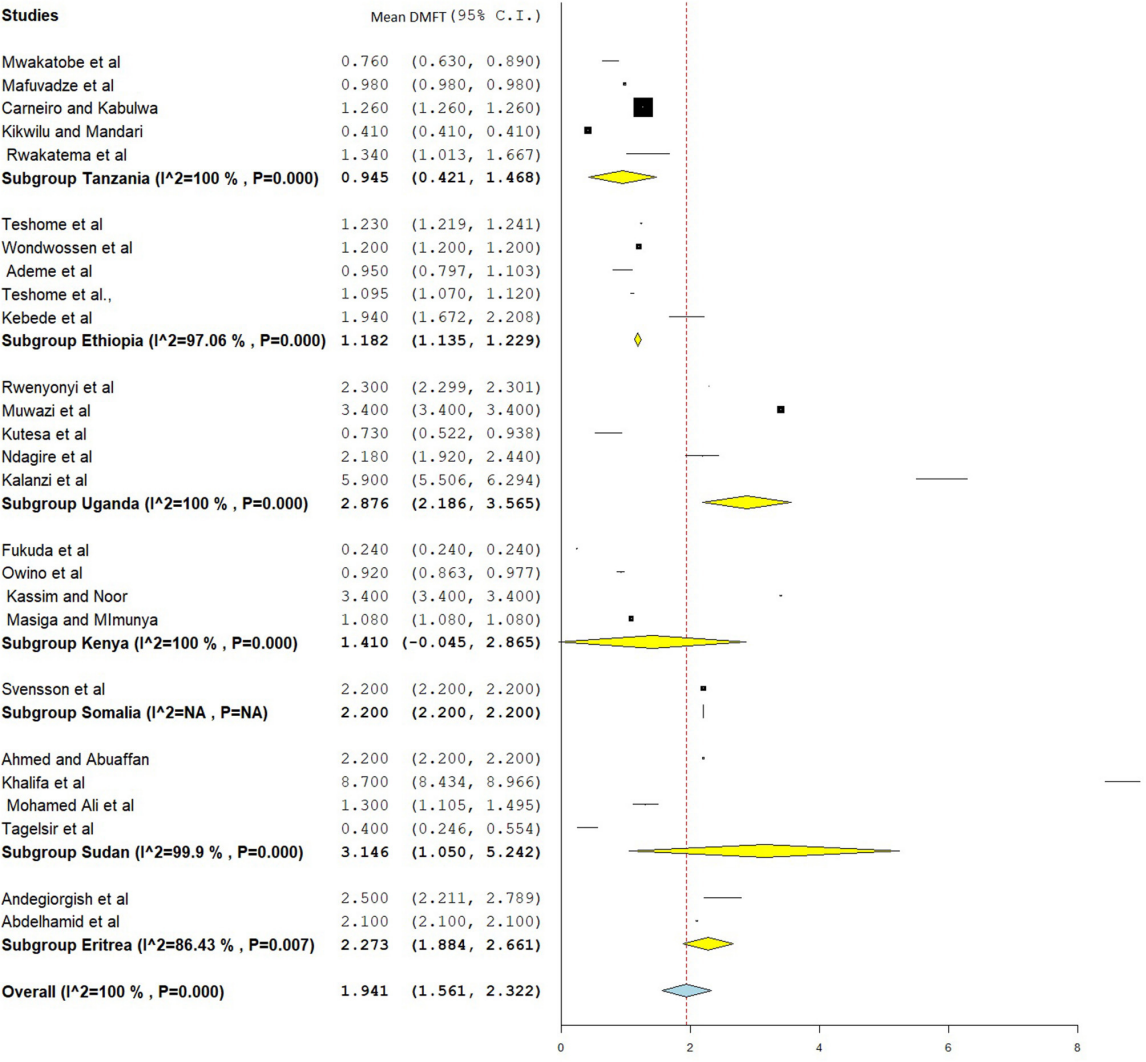
Subgroup analysis on mean DMFT based on country.

**Figure 9:**
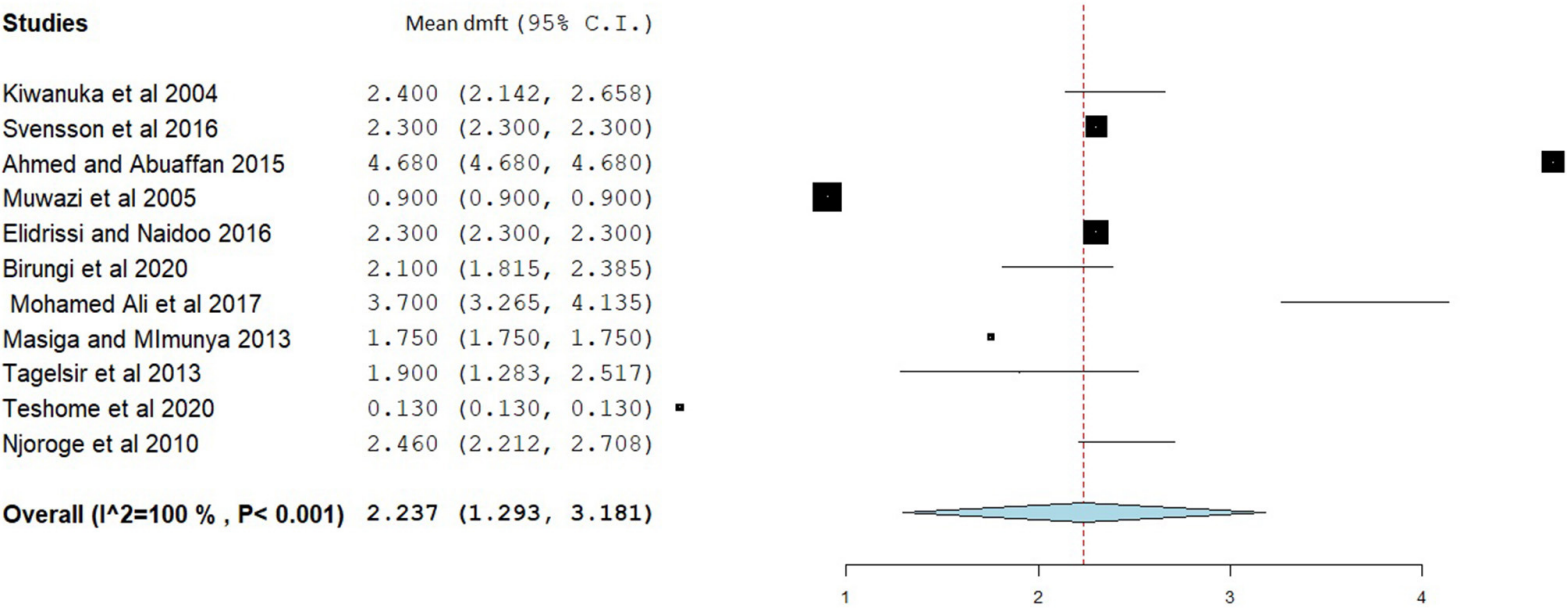
Forest plot showing the mean dmft in East Africa.

The overall mean DMFT score was 1.941 (95% CI = 1.561–2.322) (Figure 7). Subgroup analysis showed that the mean DMFT were 3.146 (95% CI = 1.050–5.242) in Sudan, 2.876 (95% CI = 2.186–3.565) in Uganda, 2.273 (95% CI = 1.884–2.661) in Eritrea, and 1.182 (95% CI = 1.135–1.229) in Ethiopia (Figure 8). Moreover, the mean decayed, missed, and filled teeth in primary dentition (dmft) were 2.237 (95% CI = 1.293–3.181) (Figure 9).

### Factors Associated With Dental Caries

In this review, carbohydrate intake, oral hygiene practice, residency, and gender were tested for association with dental caries, and a separate meta-analysis was done for each variable. A total of 11 studies were included to estimate the association between sugary food intake and dental caries, and the pooled odds ratios showed that there was a statistically significant association between carbohydrate intake and dental caries (OR = 1.575, 95% CI = 1.13–3.051).

To determine the association between gender and dental caries, 12 studies were included. The pooled odds ratios indicated that females were 1.333 times more likely to develop dental caries as compared with males (OR = 1.333, 95% CI = 1.129–1.575). Besides, the pooled ORs did not show a statistically significant association between residency and dental caries (OR = 0.962, 95% CI = 0.595–1.554).

Finally, we assessed the association between tooth brushing practice and dental caries using the random effect model. A total of 10 studies were included in the analysis, and the results revealed that those who had no tooth brushing practice were 1.967 times at risk of developing dental caries (OR = 1.967, 95% CI = 1.205–3.211) (Table 2).

**Table 2:** Risk factors of dental caries in East Africa.

**Factor (ref group)**	**Number of studies**	**Total number of participants**	**Pooled odds ratio (95% CI)**	**Test for the overall effect**	
				**I^**2**^**	**P-value**
Gender (male)	12	5,391	1.333(1.129, 1.575)	47.53	0.034
Residency (rural)	6	2,489	0.962 (0.595, 1.554)	82.3	0.067
Oral hygiene practice (good tooth brushing habit)	10	4,927	1.967 (1.205, 3.211)	85.68	<0.001
Carbohydrate intake (infrequent)	11	5,651	1.575 (1.13, 3.051)	93.38	<0.001

### Risk of Bias Within Studies

The quality of the selected studies was assessed based on the JBI critical appraisal tool (69), and studies with a quality assessment score of 50% and above were included in the review.

## Discussion

Dental caries is one of the neglected health problems in developing countries, including East African countries. Estimating the pooled prevalence and associated factors of dental caries in East Africa may contribute to informing policy makers in the region to design interventions and remedial actions. To date, there are no comprehensive data on the prevalence of dental caries and associated factors in East Africa.

The present meta-analysis revealed that 45.7% (95% CI = 38.0–53.4) of people had dental caries in East Africa, which is similar to a meta-analysis done in Ethiopia (40.98%, 95% CI = 31.62–50.34) (17) and a study done in China (41.15%) (70). However, the estimate of dental caries in the present study was lower than those in studies done in Gulf countries (64.7%) (71), Brazil (72.9%) (72), Kosovo (72.80%) (73), and China (67%, 95% CI = 56.0–77.0) (74). This difference might be due to differences in socioeconomic, dietary habits, oral hygiene practices, and knowledge and attitude of oral health prevention programs in these countries (75). In the subgroup analysis, a high prevalence of dental caries was found in Eritrea (65.2%, 95% CI = 49.2–81.1), followed by Sudan (57.8%, 95% CI = 36.0–79.7), and a low prevalence in Tanzania (30.7%, 95% CI = 21.5–39.9). This difference might be due to socioeconomic and dental facility differences between the countries in the region.

A subgroup analysis revealed that the prevalence of dental caries was age-dependent, with pooled prevalences of 39.5% (95% CI = 26.9, 52.1) in primary dentition, 41.3% (95% CI = 33.5–49.2) in mixed dentition, and 50.2% (95% CI = 38.4–62.1, *P* = 0.000) in permanent dentition. This finding is in line with a national oral health survey done in China (76) and Palestine (77), where 55.3% and 54.35% of children above 12 years had dental caries, respectively. Moreover, a meta-analysis done in Eastern Mediterranean countries found prevalences of 65% (45–85%) in primary dentition, 66% (59–73%) in mixed dentition, and 70% (64–75%) in permanent dentition (78).

The pooled estimates showed that the mean DMFT in East Africa was 1.941 (95% CI = 1.561–2.322), which is similar to studies done in India (DMFT = 1.95) (79) and Iran (DMFT = 2.33, 95% CI = 2.12–2.54) (80). However, this result is low compared to studies done in Arab League countries (DMFT = 2.469, 95% CI = 2.019–2.919) (81), Gulf countries (DMFT = 2.57) (71), and the Saudi population (DMFT = 3.34) (82). A meta-analysis in Southeast Asian countries found a mean DMFT of 0.51, which is low compared to the present study. Subgroup analysis found a high DMFT in Sudan (DMFT = 3.146) and a low DMFT in Tanzania (DMFT = 0.945). This might be due to differences in the way of life between the populations of the countries involved in the study.

This study found a mean dmft of 2.237 (95% CI = 1.293–3.181) in primary dentition, which is low compared to studies done in Saudi Arabia (dmft = 5.38, 95% CI = 4.314–6.436) (82), Gulf State countries (dmft = 5.136 ± 0.038) (71), and Arab League countries (dmft = 4.341, 95% CI = 3.714–4.969). This difference might be due to socioeconomic status differences and dietary practice differences between the countries.

The pooled analysis showed that females were 33.3% more likely to develop dental caries than males (OR = 1.333, 95% CI = 1.24–1.46), which is in line with a study done in China (42.88 vs. 39.77%) (70). The higher prevalence of caries among females might be due to the earlier eruption of teeth in girls, easier access to food supplies by women, and frequent snacking during food preparation and pregnancy (83). Moreover, consumption of carbohydrates increases the chance of developing dental caries by 1.575 times (OR = 1.575, 95% CI = 1.13–3.051), which corresponds with studies done in Ethiopia (17), Kenya (84), and Brazil (85). This might be due to the easy fermentation of carbohydrates by cariogenic bacteria into lactic acid, which facilitates the dissolution and destruction of the hard tissue of the teeth.

The present study revealed that there was a statistically significant association between poor tooth brushing habits and dental caries. Participants with poor oral hygiene practice were 1.967 times at risk of developing dental caries (OR = 1.967, 95% CI = 1.205–3.211). This is similar to a study done in Spain (OR = 1.83, 95% CI = 1.07–3.15) (86). However, the result is incomparable to a previous study done in Ethiopia (OR = 0.71, 95% CI = 0.17–2.96). This might be because few studies were included in the pooled estimates of the previous study (17). Nevertheless, the present study did not find a statistically significant association between residency and dental caries (OR = 0.962, 95% CI = 0.595–1.554), which is against the results found in Spain (OR = 1.3, 95% CI = 1.02–1.80) (86) and Ethiopia (adjusted OR = 1.6, 95% CI = 1.2–4.3) (87), where urban residents are at high risk of developing dental caries than rural residents.

## Strengths and Limitations of the Study

This study used multiple databases to search all the relevant studies for systematic review and meta-analysis. Moreover, the two reviewers, to minimize error, independently did data extraction. This was also the first meta-analysis in the region and provided baseline data on the prevalence of dental caries in East Africa. There was no language restriction.

The authors faced certain limitations during this study. Although we used a comprehensive search of articles, there is a scarcity of studies in some countries of the region. Secondly, most of the studies present the status of dental caries in terms of percentage, and only a few studies used DMFT/dmft, which is one of the indicators of the severity of the disease.

## Conclusion

The overall prevalence of dental caries was comparatively high. Being female and having poor oral health practice were independent risk factors of dental caries. The Ministry of Health of the member countries, along with dental associations of each country, ought to offer due attention to strengthen the oral health programs in schools and primary health care centers and the implementation of school water fluoridation.

## Author Contributions

AT contributed to the conceptualization, methodology, analysis, validation, writing the original draft, writing the final version, and editing. AM and BG helped with the methodology, analysis, validation, writing the original draft, writing the final version, and editing. All authors have read and approved the manuscript.

## Conflict of Interest

The authors declare that the research was conducted in the absence of any commercial or financial relationships that could be construed as a potential conflict of interest.

## Abbreviations

CI: confidence interval
CINAHL: cumulative index to nursing and allied health literature
DMFT: decayed, missed, and filled permanent tooth
dmft: decayed, missed, and filled primary tooth
EMBASE: Excerpta Medica database
JBI: Jonna Briggs Institute
PRISMA: preferred reporting items for systematic reviews and meta-analysis
SEAR: South-East Asian Region.
